# Retraction: Long noncoding RNA XIST binding to let‐7c‐5p contributes to rheumatoid arthritis through its effects on proliferation and differentiation of osteoblasts via regulation of STAT3

**DOI:** 10.1002/jcla.24160

**Published:** 2021-12-06

Retraction: Long noncoding RNA XIST binding to let‐7c‐5p contributes to rheumatoid arthritis through its effects on proliferation and differentiation of osteoblasts via regulation of STAT3 by Zong‐Qiang Wang, Dian‐Hui Xiu, Jin‐Lan Jiang, Gui‐Feng Liu, J Clin Lab Anal. 2021; 34: e23496.

The above article, published online on 03 September 2020 in Wiley Online Library (https://onlinelibrary.wiley.com/doi/10.1002/jcla.23496), has been retracted by agreement between the authors, the journal Editors in Chief Junming Guo and Rong Fu, and Wiley Periodicals LLC. The retraction has been agreed because the authors found that their published results cannot be replicated and validated by subsequent experiments. Therefore, the conclusions of the article are considered unreliable.

